# Post-cholecystectomy Gallstone Ileus: A Narrative Review

**DOI:** 10.7759/cureus.81631

**Published:** 2025-04-02

**Authors:** Ahmed Taha, Natalie Hassan

**Affiliations:** 1 General Surgery, Nottingham University Hospitals NHS Trust, Nottingham, GBR; 2 Plastic Surgery, University Hospitals of North Midlands NHS Trust, West Midlands, GBR

**Keywords:** bowel obstruction, dropped gallstone, gallstone ileus, gallstone ileus post-cholecystectomy, gallstones, pneumobilia, post-cholecystectomy, rigler´s triad

## Abstract

Gallstone ileus (GSI) is a rare cause of mechanical obstruction, caused by a gallstone that enters the gastrointestinal tract typically via a cholecystoduodenal fistula. Post-cholecystectomy gallstone ileus (PCGSI) is a specific subset occurring in patients who have previously undergone cholecystectomy. This paper aims to explore the characteristics and proposed aetiologies of PCGSI through a comprehensive narrative review of 26 case reports published between 2000 and 2024. The mechanisms leading to PCGSI are categorized into several aetiologies: bilioenteric fistulas, transpapillary gallstones, lost stones during cholecystectomy, and reconstructive surgery-associated gallstones. The findings underscore that while PCGSI shares similarities with GSI, it can present unique challenges due to its varied aetiologies, leading to misdiagnosis and consequent delays in treatment. The study emphasizes the importance of accurate imaging techniques, particularly CT scans, for effective diagnosis and highlights the need for awareness among clinicians regarding this rare condition's presentation and underlying mechanisms.

## Introduction and background

Gallstone ileus (GSI) is defined as the mechanical obstruction of the gastrointestinal tract, typically in the ileum, secondary to impaction of gallstones. GSI is a rare cause of bowel obstruction, accounting for 0.095% of total causes of bowel obstruction. Typically, the gallstone gains access to the small bowel via a cholecystoduodenal fistula, which results in physical obstruction, localised inflammation, and edema. It presents with features of bowel obstruction such as abdominal pain, abdominal distension, constipation, nausea, and vomiting.

Differential diagnoses include other causes of bowel obstruction, such as dietary constipation, adhesions, tumors, surgical ileus, and volvulus. The gold standard investigation for GSI is a CT scan of the abdomen, utilizing Rigler's triad as a radiological tool for diagnosis. The cornerstone of management for GSI is enterolithotomy primarily, with or without segmental bowel resection if ischaemic or necrotic bowel is present. The management of the fistula remains controversial due to its associated mortality but is case-dependent. GSI is associated with a mortality rate of 7% [1].

Post-cholecystectomy gallstone ileus (PCGSI) is defined similarly to GSI but occurs in patients who have undergone laparoscopic or open cholecystectomy. While data on the incidence and prevalence of PCGSI are limited, a study of 1,001 GSI cases identified six instances of PCGSI [2]. A systematic review by Meier et al. analyzed 49 cases of PCGSI over the past eight decades, underscoring its rarity [3].

PCGSI presents similarly to GSI, with typical features of bowel obstruction. It is also managed similarly, although the aetiologies of PCGSI differ from GSI and hence bilioenteric fistula may not be present. Due to its unusual nature and complex aetiology, diagnosing PCGSI can be challenging. This paper aims to synthesize the findings on PCGSI through a narrative review of the existing literature.

## Review

Methods

This narrative review encompasses 26 case reports retrieved from published literature (PubMed and Google Scholar) spanning from 2000 to 2024. The years included are due to the increased usage of CT scans as the gold standard for diagnosing both GSI and PCGSI, as well as the limited case reports published on PCGSI. The included case reports focus on patients who have previously undergone cholecystectomy and have been confirmed to have PCGSI, with confirmation achieved through surgical enterotomy. The data collected from these case reports include various parameters such as age, gender, time since cholecystectomy, symptoms, imaging modalities used, radiological findings, location of obstruction, stone size, and proposed aetiology (Table 1).

**Table 1:** Epidemiological and biological characteristics of 26 cases of PCGSI. BeF: bilioenteric fistula, TP: transpapillary stones, LS: lost stones during cholecystectomy, RSaS: reconstructive surgery-associated stones, NM: not mentioned, ERCP: endoscopic retrograde cholangiopancreatography, ES: sphincterotomy

Paper #	Year	Age	Sex	Time since cholecystectomy	Stone Size	Radiological findings	Stone aetiology	Author
1	2000	73	M	21 years	1.4 cm	BO and pneumobilia	TP post-ERCP and ES	Inglott and Williamson [4]
2	2000	74	F	15 years	3.5 cm	BO	RSaS	Wada et al. [5]
3	2003	75	M	8 years	6 cm	BO and ectopic stone	LS	Habib et al. [6]
4	2008	72	M	24 years	3.3 cm	Ectopic stone	TP	Saedon et al. [7]
5	2009	44	M	3 years	2 stones of 3 cm	BO and ectopic stone	TP	Papavramidis et al. [8]
6	2010	94	F	30 years	2.5 cm	BO and ectopic stone	BeF	Zens and Liebl [9]
7	2012	56	F	6 months	5.3 cm	BO, ectopic stone and evidence of fistula	LS	Ivanov et al. [10]
8	2012	51	M	9 days	3.5 cm	BO and ectopic stone	BeF identified	Martin et al. [11]
9	2015	83	M	40 years	NM	BO, ectopic stone and pneumobilia	BeF	Mansson and Norlen [12]
10	2017	69	F	30 years	2.8 cm	BO	BeF	Prasad et al. [13]
11	2017	92	M	30 years	5 cm	BO, ectopic stone and pneumobilia	NM (had previous ERCP and ES)	Fedele et al. [14]
12	2018	37	F	1 year	2 cm	BO, ectopic stone and pneumobilia	BeF	Teelucksingh et al. [15]
13	2019	55	F	20 years	2 cm	BO and ectopic stone	LS	Beniwal et al. [16]
14	2019	90	F	50 years	5 cm	Gastric outlet obstruction, ectopic stone and pneumobilia	BeF	Chong et al. [17]
15	2020	87	M	4 years	4 cm	BO and soft tissue mass	LS	Meier et al. [3]
16	2020	68	F	20 years	1.8 cm	BO and soft tissue mass	RSaS	Mugino et al. [18]
17	2021	80	F	6 years	6.5 cm	BO and ectopic stone	BeF	Kathawa et al. [19]
18	2021	52	M	4 days	4 cm	Ectopic stone	BeF identified	Segundo et al. [20]
19	2022	81	M	20 years	7 stones of 2 cm	BO and ectopic stone	NM	Petrillo et al. [21]
20	2022	52	M	10 years	2 stones size NM	BO, ectopic stone, pneumobilia and evidence of fistula	NM	Jamtani et al. [22]
21	2023	92	F	NM	3.2 cm	BO, ectopic stone and pneumobilia	NM	Alenezi et al. [23]
22	2023	81	M	25 years	2 cm	BO	LS	Helmy and Ryska [24]
23	2023	50	F	30 years	5.8 cm	BO, pneumobilia and intussusception	RSaS	Todd et al. [25]
24	2023	44	F	7 days	NM	Ectopic stone	BeF identified	Alsairy et al. [26]
25	2023	89	F	7 years	2.5 cm	BO and ectopic stone	LS	Wang and Goodwin [27]
26	2024	85	M	35 years	3 cm	BO	TP	Gordon et al. [28]

Patient characteristics

The age of the admitted patients ranged from 37 to 94 years, with a mean of 70 years and a median of 73.5 years. The gender distribution was equal, with 50% of cases being male and 50% female. The time since cholecystectomy varied from four days to 50 years, with a mean of 17.2 years and a median of 20 years (Table 1). The presentation of PCGSI closely resembles that of GSI or mechanical bowel obstruction. Abdominal pain was reported in 92.3% of cases, while nausea and/or vomiting occurred in 84.6%. Abdominal distention was noted in 34.6% of cases, and fever was present in 14.3%. CT scans were utilized in 100% of the cases, with additional investigations including X-rays (46.1%), ultrasound (26.9%), and magnetic resonance cholangiopancreatography (MRCP) (11.5%).

In terms of investigative features, 84% of cases exhibited signs of bowel obstruction, including bowel dilatation, transition points, air-fluid levels, and other indicators. In addition, 65.3% of cases revealed gallstones on CT scans, which included clearly visualized calcified gallstones or signs identifying gallstones, such as the Mercedes-Benz sign. In 19.2% of cases, an intraluminal mass or body was identified but not classified as a gallstone, with diagnoses including intussusception and lymphoma recurrence. Notably, 15.4% of cases did not show any intraluminal mass or gallstone within the bowel, leading to a presurgical diagnosis of GSI in 65.3% of the cases. Pneumobilia was observed on CT scans in 30.8% of cases, and among these, 37.5% indicated a bilioenteric fistula as the aetiology. In addition, 25% of these cases with pneumobilia had undergone undergone endoscopic retrograde cholangiopancreatography (ERCP) with sphincterotomy (ES) prior to the development of PCGSI (Table 1).

In terms of obstruction location, 3.8% of gallstones were found in the duodenum, 11.5% in the jejunum, 65.3% in the ileum, 3.8% in the large bowel, and 15.4% in a surgically reconstructed bowel from previous surgery, such as Roux-en-Y hepaticojejunostomy (RnYHJ). The extracted gallstones ranged in size from 1.4 to 6.5 cm, with a mean size of 3.48 cm and a median size of 3.2 cm.

**Figure 1:**
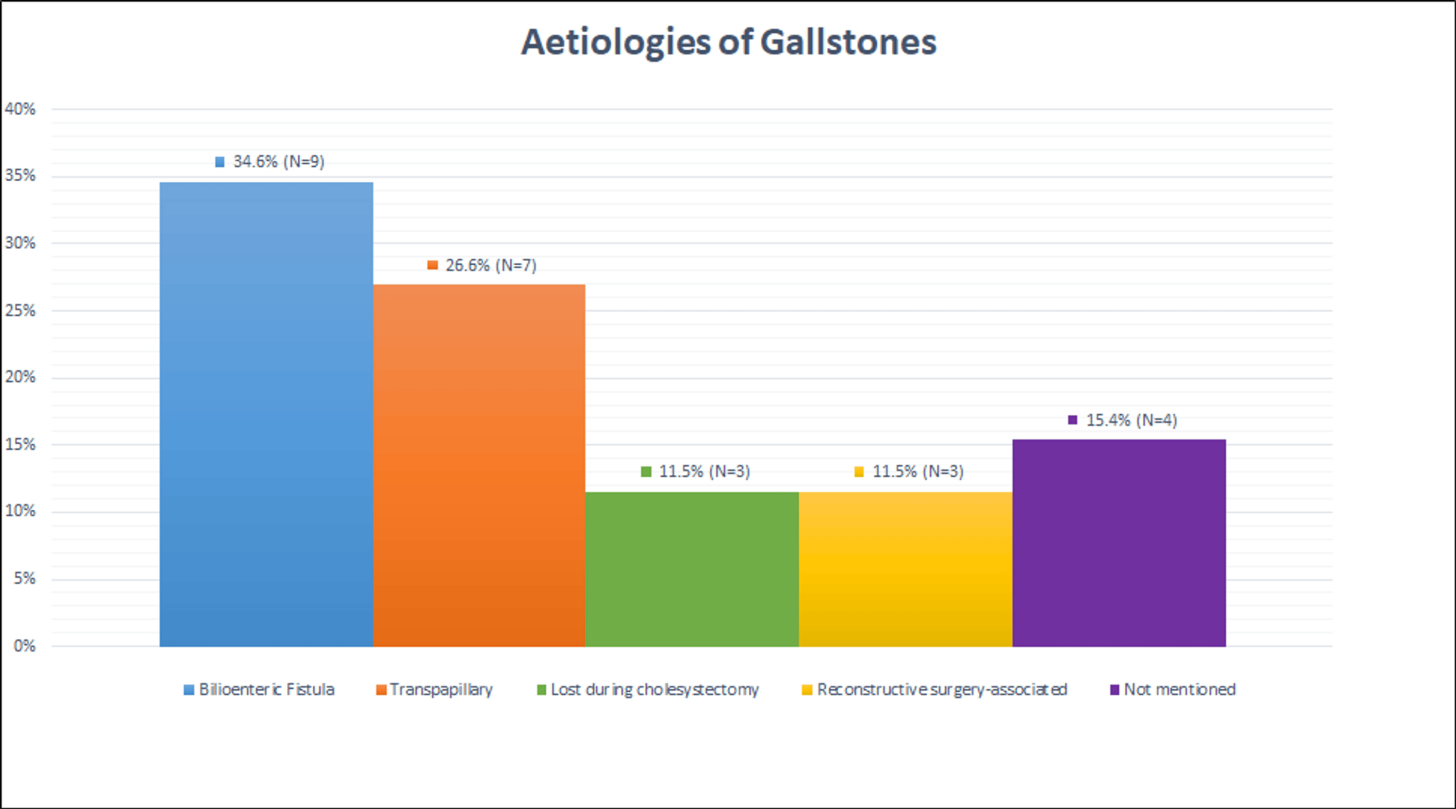
Aetiologies of gallstones in PCGSI among the 26 case reports identified within this paper.

Several mechanisms have been proposed to explain how the gallstones enter the gastrointestinal tract as outlined below (Figure 1): 1) Bilioenteric fistula: A portion (34.6%) of cases reported that the gallstone's aetiology is due to a fistula between the gallbladder and the small or large bowel. Among these, 33.3% are identified as cholecystoduodenal fistulas, 44.4% are suspected cholecystoduodenal fistulas, and 22.2% are suspected choledochoduodenal fistulas. 2) Transpapillary gallstones: Some cases (29.6%) indicated that gallstones passed through the ampulla of Vater into the bowel and remained dormant, gradually growing until leading to obstruction. 3) Lost gallstones during cholecystectomy: Some cases (11.5%) reported that gallstones spilled from the gallbladder during cholecystectomy and were not retrieved, eventually eroding into the small or large bowel from the peritoneal cavity. 4) Reconstructive surgery-associated gallstones: Some cases (11.5%) involved patients with previous surgeries that predisposed them to developing gallstones within surgically modified bowel, such as in RnYHJ. 5) Not mentioned: Some cases (15.4%) did not specify a mechanism for how the gallstone entered the bowel. 19% of cases suggested that small bowel or colonic diverticula may have played a role in harbouring the gallstone, allowing it to grow over time before dislodging and causing PCGSI. Overall, 46% of all cases had their aetiology confirmed, with either a fistula identified and repaired, or a gallstone seen within the peritoneum before admission with PCGSI.

Discussion

Typically, the progression leading to GSI involves several key steps: the onset of recurrent or severe cholecystitis, the formation of a bilioenteric fistula (most commonly a cholecystoduodenal fistula), followed by the translocation of a gallstone from the gallbladder into the bowel, ultimately resulting in bowel obstruction [29]. However, various alternative scenarios can arise at each stage. For instance, different types of fistulas may form, such as choledochojejunal fistulas; there can also be gastric pylorus obstruction, known as Bouveret’s syndrome; or even obstruction of the large bowel, leading to gallstone coleus [30].

The presentation of PCGSI resembles that of GSI, typically featuring colicky abdominal pain, nausea, vomiting, and abdominal distention, with fever observed in a few cases. Some cases exhibit the "tumbling" phenomenon, where obstructive symptoms resolve spontaneously without intervention due to the intraluminal gallstone temporarily dislodging and re-lodging before complete obstruction occurs [3,29]. This phenomenon can complicate management if misdiagnosed as adhesional small bowel obstruction, leading to inappropriate conservative treatment. In addition, it may delay patients seeking medical attention due to the intermittent nature of symptoms, as the stone moves through the gastrointestinal tract.

The pathophysiology of PCGSI may share similarities to GSI, yet it can differ significantly. For instance, stones may have passed through the ampulla of Vater prior to the cholecystectomy, remaining dormant in the bowel for decades and gradually growing until they eventually lead to GSI. Alternatively, stones that were lost during the cholecystectomy may drop into the peritoneal cavity and subsequently erode into the bowel lumen, resulting in GSI.

Most gallstones entering the gastrointestinal tract are excreted in stool, but stones larger than 2-2.5 cm often cause mechanical bowel obstruction. In GSI, obstructing stones average 4.3 cm in size [29,30]. This paper's findings align with this trend, reporting an average stone size of 3.48 cm in obstructing cases, supporting the correlation between larger stones and obstruction risk.

The ileum is the most common site of impaction in PCGSI, similar to GSI, due to the progressively narrowing lumen of the small bowel [24,31]. Chong et al. reported a rare case of Bouveret's syndrome in a post-cholecystectomy patient, where a gallstone obstructed the gastric pylorus, causing gastric outlet obstruction. This was notable as the first instance of Bouveret's syndrome in a patient without a gallbladder [17]. Ivanov et al. described another PCGSI case involving a lost gallstone that partially eroded into the right angle of the transverse colon six months post-cholecystectomy, leading to subocclusive gallstone coleus, whilst also forming a cutaneous fistula towards the right flank [10].

Radiological Features

Rigler's triad, the pathognomonic diagnostic criteria for GSI, includes pneumobilia, ectopic gallstone, and signs of bowel obstruction. The presence of two out of three signs is considered a positive Rigler's triad. Notably, researchers have expanded this to Rigler's tetrad by adding the change in location of a previously noted gallstone and further to Rigler's pentad by including a dual air-fluid level in the right upper quadrant of the gallbladder [30,32].

Ultrasound, the first-line investigation for gallbladder pathology, is mostly limited to detecting gallbladder and proximal common bile duct stones due to bowel gas interference [33]. X-rays are now less favoured, as only 15-20% of gallstones are radiopaque [33], and Rigler's triad is identified in just 4-53% of cases [30], limiting their diagnostic value for PCGSI. MRCP, used in 11.5% of cases, provides advantages over CT scans by detecting isoattenuating gallstones relative to bile or fluid, making it useful as a second-line investigation when initial results are unclear [29]. Contrast-enhanced CT scans are the most effective diagnostic tool for GSI, with a sensitivity of 93%, specificity of 100%, and accuracy of 99% [34]. They serve as the first-line investigation for bowel obstruction in suspected cases.

In a comparative analysis, Lassandro et al. found that CT scanning significantly outperformed other imaging modalities in detecting Rigler's triad, with a detection rate of 77.8%, markedly higher than X-rays (14.8%) and ultrasound (11.1%). In the study, the most common CT findings were bowel loops dilatation (92.6% of cases), followed by pneumobilia (88.9%), ectopic gallstone (81.5%), air-fluid levels (37%), and biliodigestive fistula (14.8%) [29].Detecting gallstones on CT scans is influenced by factors such as stone size, superficial calcification, composition, and location relative to surrounding tissue, which can lead to isoattenuation with bile or fluid [30]. In this study, 65.3% of the cases identified the offending gallstone, resulting in an accurate pre-laparotomy diagnosis.

**Figure 2:**
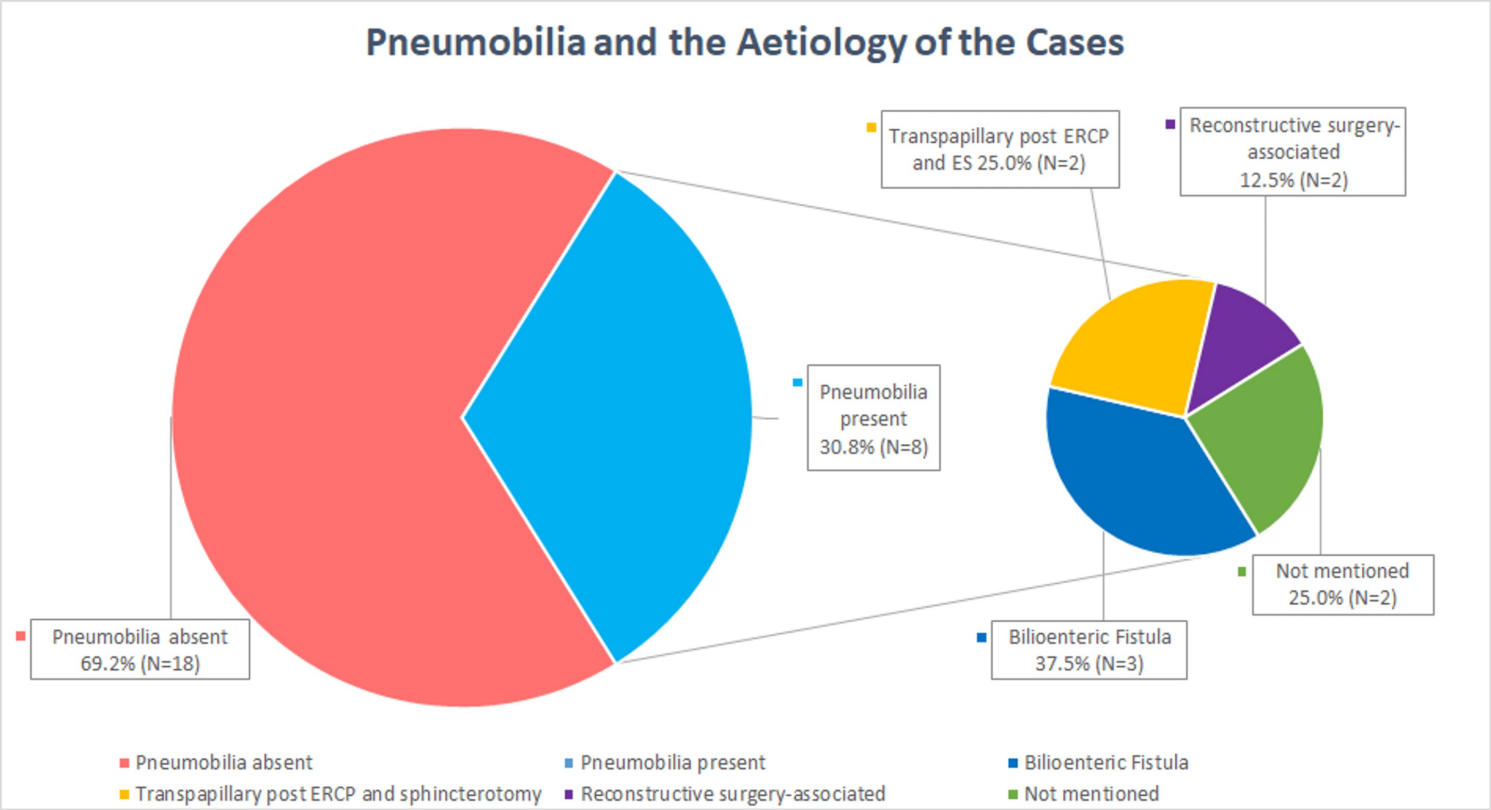
Prevalence of pneumobilia and its associated aetiologies among the 26 case reports identified within this paper.

Pneumobilia was detected in only 30.8% (N = 8) of cases in this study of PCGSI, compared to 88.9% in GSI (Figure 2). This discrepancy may be attributed to the lower prevalence of bilioenteric fistulas in PCGSI relative to GSI. In over 90% of GSI cases, gallstones enter the gastrointestinal tract via a bilioenteric fistula, with cholecystoduodenal fistulas being the most common. This results in the presence of pneumobilia, which can be identified on CT scans. In PCGSI, 34.6% (N = 9) of cases suggested a suspected or identified bilioenteric fistula as the aetiology; among these, five cases exhibited pneumobilia on CT. In addition, patients with active bilioenteric fistulas or those who have undergone surgical reconstructions (e.g., RnYHJ, choledochojejunostomy bypass) are likely to present with pneumobilia on CT scans [25,35]. However, patients with lost stones during cholecystectomy or transpapillary stones that migrate into the bowel would not demonstrate pneumobilia. Thus, of the 22 cases with suggested aetiologies, 54% (N = 12) could theoretically present with pneumobilia, assuming accurate aetiological assessments. 

Pneumobilia as a radiological sign, although useful in the diagnosis of GSI, may be more of a hinderance in PCGSI. In addition to the reasons above, pneumobilia can arise from several cause including bilioenteric surgical anastomosis, bilioenteric fistula, recent biliary instrumentation, ERCP with ES, infections, and rarely blunt trauma [36]. Among the eight cases with pneumobilia on CT scan, 37.5% (N = 3) reported a bilioenteric fistula as the aetiology. In addition, 25% (N = 2) of these cases had undergone ERCP with ES prior to PCGSI presentation, which would lead to pneumobilia [36]. One of the cases proposed that the stone likely dropped into the duodenum via the ampulla of Vater post-ERCP and ES, while the other case had no suggested aetiology (Figure 2).

The literature reports spontaneous fistula closure in 61.5% of cases of GSI, without specifying timeframes [31]. The average interval between cholecystectomy and presentation in this study is 16.5 years. This suggests that the fistulas likely formed pre-cholecystectomy, potentially healing after allowing gallstone passage into the gastrointestinal tract. These stones could remain dormant for years before causing obstruction. Consequently, neither pneumobilia nor fistulas may be detectable on admission due to spontaneous closure of the fistula and resolution of the pneumobilia. Of the nine cases suggesting bilioenteric fistula aetiology, only three had identifiable fistulas, all presenting within 10 days post-cholecystectomy. The other six cases had cholecystectomies 1-50 years prior. This pattern aligns with spontaneous fistula closure over time, explaining why fistulas are often undetectable in cases with longer intervals between surgery and symptom onset.

While Rigler's triad is useful for diagnosing GSI, its relevance is diminished in PCGSI. Negative CT findings should not exclude PCGSI, given the varied aetiologies and radiological signs [25]. Clinicians must maintain a high suspicion for PCGSI in patients with a history of cholecystectomy, even when imaging results are negative.

Proposed Mechanisms

Bilioenteric fistulas are the primary cause of GSI and PCGSI, as evidenced in this paper. In GSI, these fistulas manifest in various forms, with cholecystoduodenal fistulas being the most prevalent (32.5-96.5%). Other types include cholecystogastric (0-13.3%), choledochoduodenal (0-13.4%), cholecystocolic (0-10.9%), cholecystojejunal (0-2.5%), and cholecystoileal (0-2.5%) fistulas. In some cases (0-65%), the specific type remains undetermined [29]. Unorthodox cases can occur in PCGSI. Teelucksingh et al. described a case involving a partial cholecystectomy that led to stump cholecystitis, resulting in a bilioenteric fistula post-cholecystectomy and subsequently PCGSI [15]. Mansson et al. reported a case of PCGSI 40 years after cholecystectomy, with pneumobilia observed on CT. They attributed the aetiology to a primary common bile duct stone passing through a choledochoduodenal fistula that formed after cholecystectomy [12]. These cases highlight the need to consider PCGSI in patients with a history of cholecystectomy, even decades later.

Transpapillary gallstones are the second most common cause of PCGSI, where small gallstones enter the bowels via the ampulla of Vater, lodge themselves within the bowel lumen, lay dormant while growing, and eventually obstruct the lumen. Stones smaller than 5 mm can typically pass through the common bile duct into the duodenum, with larger stones passing if ES was performed during ERCP [37]. While stones up to 2 cm can usually traverse the terminal ileum, those exceeding 2.5 cm are more likely to become lodged and remain dormant [38]. Factors influencing impaction include stone size, number, shape (cylindrical or faceted stones being more prone to impaction), bowel anatomy, and the presence of diverticula or intestinal strictures [8, 26, 29]. Inglott et al. described a case of PCGSI that developed soon after ERCP with ES. The obstruction was caused by a 1.4 cm gallstone lodged in the ileum due to a fibrotic stricture and intestinal kinking from adhesions [4].

Lost stones during cholecystectomy accounted for 11.5% (N = 3) of cases in this paper. Woodfield et al. reported gallstone spillage occurring in 7.3% of laparoscopic cholecystectomies, with 2.4% having unretrievable peritoneal stones [39]. Peritoneal gallstones act as foreign bodies, triggering localized inflammation, particularly if infected. This can lead to complications such as granulomas, abscesses, fistulas, and erosion into adjacent structures. In Western countries, fewer than 20% of gallstones are pigmented, but 80-90% of these contain bacteria, with 90% of spilled gallstones involved in peritoneal complications being pigmented, highlighting the impact that bacterial growth has on the stone's erosive capabilities [39]. Ivanov et al. described a PCGSI case where a lost gallstone during cholecystectomy partially occluded the ascending colon, leading to gallstone coleus, while forming a cutaneous fistula towards the right flank [10].

Reconstructive surgery-associated gallstones as an aetiology is scarcely reported within the literature, given the rarity of PCGSI itself. This paper presents three cases where specific surgical procedures altering biliary tree and gastrointestinal tract anatomy are hypothesized to contribute to PCGSI development [5,18,25]. The surgeries involved are total gastrectomy with Roux-en-Y reconstruction (TGRnY), pylorus-preserving pancreatoduodenectomy (PPPD) with single-loop Billroth II gastrojejunostomy reconstruction, and RnYHJ. In all three surgeries, bile is secreted into a segment of the bowel that eventually either connects to an anastomosed segment of bowel containing chyme or to the gastric pylorus to receive chyme directly. The anatomical name of this segment of the bowel receiving bile is named differently in each surgery but will be termed biliary limb for the purposes of this paper only. All three case reports speculate that gallstones formed within the biliary limb, with several hypothesized reasons: First, the biliary limb acts as a "neo gallbladder," facilitating the concentration of bile [18]. Second, bile stasis within this limb, surrounded by a cholesterol-rich environment, promotes stone formation [5]. In addition, reduced intestinal motility resulting from extensive lymphadenectomy of the segmented duodenal bowel-particularly in procedures like PPPD and Whipple's-may further contribute to this issue [18]. Lastly, lithogenic foreign material, typically remnants from previous surgeries such as sutures or stents, can serve as a focal point or nidus for gallstone formation within the biliary limb, promoting crystallization [25].

Jejunal limb obstruction (surgeries with Billroth II reconstruction or Roux-en-Y reconstruction) and afferent loop syndrome (surgeries with single-loop Billroth II reconstruction) are serious complications. Obstruction of the biliary limb can lead to backflow of bile and pancreatic enzymes, resulting in ascending cholangitis or pancreatitis [40].

Management

The management of both PCGSI and GSI is similar, but the surgical treatment of GSI remains controversial due to a lack of clear consensus on the best approach. In GSI, treatment would entail either simple enterolithotomy, one-stage procedure involving enterolithotomy, cholecystectomy and fistula repair or two-stage procedure of enterolithotomy first, followed by cholecystectomy and fistula repair later. Segmental bowel resection may accompany simple enterolithotomy if ischaemic or perforated bowel is present. The choice of surgery depends on many factors that influence both morbidity and mortality [29]. In a 1994 study by Reisner and Cohen, simple enterolithotomy had a mortality rate of 11.7%, while the more invasive one-stage surgery showed a higher mortality rate of 16.9% [31]. Spontaneous closure of biliary fistulas occurs in 61.5% of cases of GSI. Conversely, persistent bilioenteric fistulas can lead to serious complications such as retrograde cholecystitis, recurrent GSI, and gallbladder cancer. Notably, 86.7% of recurrent GSI cases have involved untreated bilioenteric fistulas. In addition, 11% of cholecystoduodenal fistulas and 60% of cholecystocolonic fistulas have resulted in cholangitis, while 15% of fistula cases have shown complications related to gallbladder cancer [31]. Enterolithotomy is generally preferred for most patients; one-stage procedures may suit low-risk, stable patients; and two-stage surgery is considered for those with persistent symptoms after enterolithotomy [38].

## Conclusions

In summary, PCGSI is an exceedingly rare disease with an elusive diagnosis. PCGSI typically presents similarly to GSI and is equally treated, but the diagnosis can be challenging given the rarity of the condition. Furthermore, Rigler’s triad would not be as reliable in diagnosing PCGSI as it would be in GSI. Most cases are suspected to be due to a bilioenteric fistula. Early presentation from the date of cholecystectomy alludes toward a patent bilioenteric fistula but may present many years later with a variety of aetiology. Further research is required to understand the aetiology of PCGSI and formulate a more effective diagnostic criteria.
